# Transforming Health Insurance in Bangladesh: A Future‐Ready Approach

**DOI:** 10.1002/hsr2.70945

**Published:** 2025-06-20

**Authors:** MD. Faisal Ahmed

**Affiliations:** ^1^ Department of Health Sciences and Informatics Bangladesh Institute of Innovative Health Research Mirpur Dhaka Bangladesh

## Author Contributions

**MD. Faisal Ahmed:** writing – review and editing, writing – original draft, conceptualization, methodology, investigation, validation, resources, data curation.

## Conflicts of Interest

The author declares no conflicts of interest.

Dear Editor,

I am writing in response to *“The Urgent Need for Developing a Common Health Insurance Policy in Bangladesh: A Perspective”* [1]. The article effectively highlights the pressing need for a universal health insurance system in Bangladesh. However, it largely advocates for conventional solutions that have faced challenges in implementation across low‐ and middle‐income countries. A paradigm shift is necessary—one that moves beyond traditional state‐led models to explore decentralized, technology‐driven, and behaviorally informed strategies tailored to Bangladesh's economic and social realities.

Habib and Molla reports that out‐of‐pocket healthcare spending amounts to 68.5% of total healthcare costs in Bangladesh which causes financial difficulties for numerous citizens [2]. They suggest raising government funding while improving coverage through existing health programs. Using only state‐funded initiatives fails to address the constraints that stem from both fiscal capacity and administrative efficiency issues. A better solution combines mandatory insurance with voluntary options through digital financial access while employing behavioral economics to boost participation rates.

The scarcity of health insurance payments stems from people's distrust of financial institutions and their inability to see immediate advantages from coverage. Behavioral economics provides solutions through default enrollment models which require people to actively decline insurance coverage. Mobile banking platforms bKash and Nagad should integrate health insurance services through automatic micro‐premium withdrawals which maintain user involvement while avoiding yearly payment requirements. Insurance communication becomes more effective through behavioral alignment when risk protection messages replace long‐term health investment messaging.

A new approach would be the implementation of health insurance models supported by diaspora communities. The annual remittance amount of over $22 billion in Bangladesh lacks an organized system to direct this money toward healthcare funding. Insurance plans that allow expatriates to pay insurance premiums for family members and support community‐based risk funds would enhance healthcare coverage among vulnerable populations. The Philippines and Mexico together with other countries have established successful diaspora‐backed healthcare insurance systems which reduced healthcare expenses paid directly by patients to millions of people [3, 4].

Technological integration is also crucial. The article correctly identifies healthcare financing problems yet fails to investigate blockchain‐based claims automation and AI‐based adaptive pricing solutions. Blockchain technology brings transparency to operations while reducing fraud and streamlines claim settlements through automation to establish system‐wide trust [5]. AI‐driven underwriting systems allow for risk‐based premium adjustments which enables insurance affordability for different income groups according to Rix [6]. Proof‐of‐concept deployments in Rwanda and Kenya show that these innovations can scale up for emerging markets [7, 8].

Bangladesh needs to prevent implementing models from high‐income countries because their economic and institutional structures differ too much from its own. The country needs to prioritize a combined method that includes public‐private collaborations with technological enhancements and behavioral economic practices. The implementation of mobile‐based microinsurance and remittance‐backed financing and blockchain claims processing requires initial testing through pilot programs for framework scalability purposes. The absence of forward‐thinking strategies makes universal health coverage efforts stay theoretical instead of becoming practical initiatives.

## Data Availability

Data sharing not applicable to this article as no data sets were generated or analyzed during the current study. No new data were generated or analyzed in this study. All supporting information and references are publicly available as cited in the article.
